# Mixed Forecast of Air Quality Index with a Bibranch Parallel Architecture Considering Seasonal Heterogeneity

**DOI:** 10.3390/e28040419

**Published:** 2026-04-09

**Authors:** Huibin Zeng, Ying Liu, Hongbin Dai, Xue Zhao, Ning Tian

**Affiliations:** 1 School of Economics and Management, Chongqing Normal University, Chongqing 401331, China; zenghuibin@cqnu.edu.cn (H.Z.); 2023051508048@stu.cqnu.edu.cn (Y.L.); 2 College of Computer and Information Science, Chongqing Normal University, Chongqing 401331, China; 3 School of Management, Xi’an University of Architecture and Technology, Xi’an 710055, China; zhaoxue1018@xauat.edu.cn (X.Z.); tning4891@xauat.edu.cn (N.T.)

**Keywords:** AQI prediction, interpretability, learnable fusion layer, dual-branch architecture, SHAP

## Abstract

Accurate prediction of the air quality index (AQI) is crucial for understanding urban pollution dynamics and protecting public health. This study proposes a dual-branch fusion framework (CL-XGB-Season) to address seasonal heterogeneity in AQI prediction by integrating temporal dynamic features and static patterns. The CNN-LSTM branch captures short-term temporal fluctuations, while a seasonally split XGBoost branch fits long-term static patterns via independent submodels for spring, summer, autumn, and winter. SHAP-based interpretability analysis revealed the dominant drivers across different seasons: the “temperature × O_3_” interaction feature plays a key role in summer, characterizing the ozone formation mechanism dominated by photochemical reactions under conditions of high temperature and strong solar radiation; whereas the PM_2.5_/PM_10_ ratio is crucial in winter (where pollution is primarily driven by pollutant accumulation). The dual-branch fusion framework was validated using hourly resolution data from Chongqing for the 2020–2025 period. Results indicate that the framework achieved a prediction accuracy of 0.197 root mean square error (nRMSE) and 0.9611 coefficient of determination (R^2^) on the test set, outperforming eight ablation variants and five baseline models (ARIMA, Transformer, etc.) in comparative experiments. Ablation studies confirm the necessity of dual branches and seasonal modeling, with the full model reducing nRMSE by 19–63% versus single-model variants. This framework maintains stable seasonal performance and provides actionable insights for targeted air quality management.

## 1. Introduction

With the rapid advancement of global industrialization and urbanization, atmospheric pollutants generated by industrial production, vehicle exhaust emissions, and fuel combustion have steadily increased [1]. Air pollution has emerged as a severe environmental challenge, profoundly impacting public health, ecological integrity, and socioeconomic development [1,2]. It severely damages the environment, contributing to global warming, airborne diseases, chronic health conditions, and rising cancer rates. Approximately 7 million premature deaths annually are attributed to air pollution, as reported by the World Health Organization, highlighting the severity of this issue [3]. Elevated concentrations of pollutants such as fine particulate matter (PM_2.5_), nitrogen dioxide (NO_2_), and ozone (O_3_) not only contribute to high incidence rates of respiratory and cardiovascular diseases [4,5,6] but also diminish overall urban livability and residents’ quality of life.

The Air Quality Index (AQI) serves as a crucial indicator for measuring air pollution levels. By quantitatively describing air quality conditions, it provides the public with an intuitive health risk warning. Higher AQI values indicate more severe air pollution [7] and pose greater threats to public health. Therefore, accurately predicting the AQI is of significant importance for formulating effective air quality improvement measures, safeguarding public health, and promoting sustainable economic development [8].

From a policy perspective, AQI forecasting serves as a critical basis for governments to implement environmental management and formulate policies. By anticipating future air quality trends, governments can plan more effective traffic management, industrial layout, and environmental policies to reduce pollutant emissions and improve air quality [9]. For instance, during periods with high predicted AQI values, governments can implement traffic restrictions to reduce vehicle exhaust emissions or require industrial enterprises to scale back production activities to lower pollutant discharges. Simultaneously, AQI forecasts provide vital health guidance to the public, helping individuals take necessary protective measures to mitigate the health risks associated with air pollution [9].

Chongqing’s unique geographical and climatic conditions, coupled with its complex distribution of pollution sources, make Air Quality Index (AQI) forecasting particularly critical. This mountain city, situated along the upper reaches of the Yangtze River, features predominantly hilly and mountainous terrain. This distinctive topography restricts the dispersion of atmospheric pollutants, especially during stable weather conditions when pollutants tend to accumulate. In summer, Chongqing’s hot and humid environment, coupled with strong solar radiation, facilitates photochemical reactions between volatile organic compounds (VOCs) and nitrogen oxides (NO_x_) in the atmosphere, generating photochemical smog characterized by ozone (O_3_) and leading to a significant increase in ozone concentrations; In winter, atmospheric dispersion capacity weakens under stable weather conditions, causing primary pollutants such as fine particulate matter (PM_2.5_) and nitrogen dioxide (NO_2_) to accumulate near the ground, resulting in haze and smog. These seasonal variations, driven by different dominant mechanisms, place higher demands on AQI forecasting; models must be capable of identifying and adapting to the patterns of pollution formation across different seasons. Furthermore, as a major economic hub in western China, Chongqing’s air quality impacts not only the health and quality of life of its residents but also significantly influences air quality in surrounding regions. Therefore, accurate AQI forecasting can provide scientific decision support for Chongqing and its neighboring areas, promoting overall regional air quality improvement.

The rise in deep learning technologies has provided novel solutions for AQI prediction, particularly recurrent neural networks (RNNs) [10,11] and their variants (such as LSTM and GRU) [12], which excel at handling time series data [6]. By incorporating gating mechanisms and memory units, these models effectively capture long-term dependencies within time series data, enhancing prediction accuracy while capturing dynamic variation characteristics [13]. Machine learning models like Support Vector Machines (SVM), Random Forests (RF), and Gradient Boosted Trees (GBDT) excel at handling nonlinear data and feature selection by uncovering latent patterns within data [14]. To further enhance AQI prediction accuracy, researchers have explored hybrid models. By combining the strengths of different models, hybrid approaches demonstrate superior capabilities in feature extraction, pattern recognition, and predictive precision. For instance, the CNN-LSTM hybrid model integrates the feature extraction strengths of Convolutional Neural Networks (CNN) with the time series forecasting capabilities of LSTM, achieving high-precision AQI predictions [15]. Additionally, some researchers have attempted to incorporate attention mechanisms into hybrid models to enhance the model’s focus on important features, thereby improving predictive performance [16,17,18].

Despite extensive research dedicated to AQI prediction, traditional methods and classical machine learning models still exhibit limitations in handling complex nonlinear relationships and long-term temporal dependencies [19,20].

Air Quality Index (AQI) forecasting involves monitoring data on multiple air pollutant concentrations, which exhibit high complexity and uncertainty. Simultaneously, pollutant concentrations are influenced by multiple factors, including meteorological conditions (such as temperature, humidity, wind speed, and wind direction), traffic flow, industrial emissions, and geographical environment. This results in data exhibiting nonlinear, non-stationary, and highly correlated characteristics [9]. Such complexity increases the difficulty of AQI prediction, requiring predictive models to possess strong data adaptability and feature extraction capabilities [6]. Second, air pollution levels exhibit pronounced seasonal variations. Winter often sees worsened pollution due to increased heating demands and meteorological conditions that hinder pollutant dispersion, whereas summer typically features better air quality owing to higher precipitation and meteorological conditions that facilitate pollutant dilution [21]. This seasonal pattern necessitates AQI prediction models capable of capturing and adapting to pollutant concentration variations across different seasons to enhance forecasting accuracy [22]. Furthermore, AQI data represent a classic time series with temporal dependency and trend characteristics. Pollutant concentrations evolve over time, and historical data significantly influence future predictions [13]. Consequently, models must effectively process time series data to capture dynamic features including long-term trends, seasonal fluctuations, and short-term anomalies.

To address these challenges, this study proposes an innovative “Dual-Branch Dynamic Adaptation-Seasonal Awareness Fusion Framework.” This framework employs a multi-modal feature decoupling and dynamic weight allocation mechanism. The CNN-LSTM branch captures short-term temporal fluctuations, while the XGBoost branch fits long-term static patterns. By implementing seasonal modeling, the framework enhances the model’s adaptability to seasonal variations. The authors’ main contributions are:

(1) This study proposes a novel dual-branch fusion forecasting paradigm. It represents the first instance of deeply integrating a CNN-LSTM network—used to capture temporal dynamic dependencies—with a seasonally adjusted XGBoost model—used to fit static nonlinear relationships—through a learnable nonlinear fusion layer. Comparative experiments against State-of-the-Art methods (ARIMA, Transformer, LightGBM, etc.) demonstrate that this paradigm achieves superior performance in key metrics such as RMSE and MAE for AQI forecasting.
(2) The seasonal modeling strategy within the static feature branches specifically addresses the issue of poor generalization caused by seasonal heterogeneity in the model. By training independent XGBoost models for spring, summer, autumn, and winter, respectively, the model can accurately learn the distinct physical and chemical patterns of pollution across different seasons. This significantly enhances the model’s overall adaptability to complex pollution patterns throughout the year and improves its predictive stability.
(3) The fusion design based on feature concatenation and ensemble learning effectively synergizes temporal and static information. This approach concatenates the high-dimensional feature vectors from the temporal branch with the predicted values from the static branch, then trains an independent XGBoost regressor to learn the complex mapping from these concatenated features to the final AQI. Ablation experiments confirm that this fusion approach, which incorporates a nonlinear ensemble model, significantly outperforms simpler fusion methods—such as fixed weights or linear regression—in capturing feature interactions and enhancing final prediction accuracy.

## 2. Literature Review

As a core research area in global environmental governance and public health protection, AQI forecasting has developed into a diverse and mature technical framework, with a vast body of literature exploring various modeling logics and input features. Based on the core technical approaches and modeling principles employed, existing forecasting methods can be clearly categorized into four major types: statistical models, traditional machine learning models, deep learning models, and hybrid models. From the perspective of input features, these studies can be further categorized into pure time-series modeling, static feature modeling, and multi-source feature fusion.

In the realm of pure time-series modeling, models rely entirely on historical pollutant concentration sequences for prediction, aiming to uncover the intrinsic evolution patterns of the time series. This category primarily encompasses two types of models: statistical models and deep learning models. Statistical models represent the earliest explorations in the field of AQI forecasting; typical examples include ARIMA and its variants. By modeling the autocorrelation and moving average characteristics of historical data, they achieve linear predictions of future values; however, their ability to fit nonlinear and non-stationary sequences is limited. The rise in deep learning models has provided new solutions for time series forecasting, particularly Recurrent Neural Networks (RNNs) and their variants (such as LSTM and GRU). By introducing gating mechanisms and memory units, these models can effectively capture long-term dependencies in time series data. Yang et al. [23] used a cascaded model combining a Temporal Convolutional Network (TCN) and a Bidirectional Gated Recurrent Unit (BiGRU). Taking historical AQI and pollutant concentration sequences as input, the TCN extracts multiscale temporal features, while the BiGRU captures bidirectional dependencies, significantly improving prediction accuracy. Zhou et al. [24] constructed a CNN-GRU model, using historical pollutant concentration sequences as input. The CNN extracts local correlation features, while the GRU models temporal dependencies. Sarkar et al. [25] combined LSTM and GRU deep learning models, also using only historical AQI sequences as input, to predict environmental AQI. Gilik et al. [26] proposed a CNN-LSTM hybrid deep learning model, employing both univariate (historical AQI data only) and multivariate (historical data for multiple pollutants) temporal input strategies to predict urban air pollutant concentrations. Wang et al. [27] introduced an attention mechanism into the LSTM, using historical pollutant sequences as input to enhance the capture of key temporal information. Such studies have fully demonstrated the effectiveness of deep temporal models in uncovering long-term dependencies in AQI data; however, they completely lack consideration of external static features such as meteorological conditions and human activities, resulting in insufficient predictive capability for sudden changes in pollution levels under extreme weather or emergency events.

In the field of static feature modeling, models primarily rely on pollutant concentrations, meteorological factors, and temporal features at the current time or in the recent past to make predictions, with the aim of fitting the nonlinear mapping relationships between variables. This category is dominated by traditional machine learning models, with Support Vector Machines (SVM), Random Forests (RF), and Gradient Boosted Trees (GBDT, including XGBoost and LightGBM) widely used for AQI forecasting. The input features for these models typically include structured data such as pollutant concentrations (PM_2.5_, PM_10_, NO_2_, O_3_), meteorological factors (temperature, precipitation, wind speed, humidity), and temporal features (hour, season). By uncovering the nonlinear mapping relationships between features and the target variable, these models perform exceptionally well in handling multi-source, heterogeneous data. However, traditional machine learning models inherently lack the ability to model the forward and backward dependencies in time series data; therefore, they often require feature engineering (such as constructing lagged features via sliding windows) to indirectly incorporate temporal information. To address this limitation, some studies have begun combining static features with time series data. For example, in a hybrid model combining Empirical Exponential Mode Decomposition (EEMD) and Gated Recurrent Units (GRU), Huang et al. [9] introduced 18 pollutant and meteorological variables as input features in addition to pollutant time-series data. After noise reduction via EEMD, the GRU was used to capture temporal dependencies. Mao et al. [28] adopted a feature blending strategy, concatenating pollutant time series, meteorological observations, and temporal features (such as hours) before inputting them into a time-shifting LSTM model (TS-LSTME) for multi-step forecasting. Qian et al. [29] proposed an evolutionary deep learning model that integrates XGBoost feature selection, using XGBoost to screen core pollutant and meteorological features from the raw data before feeding them into subsequent time-series models. While such studies enrich the input information by introducing static features, they mostly employ single-method fusion approaches such as “feature concatenation” or “front-end filtering,” and have not yet established specialized parallel modeling for both temporal dynamics and static patterns.

In the field of multi-source feature fusion, models aim to deeply integrate temporal dynamic information with static patterns to leverage the complementary strengths of different models. This field centers on hybrid models, which combine the strengths of various models to demonstrate superior capabilities in feature extraction, pattern recognition, and prediction accuracy. Nguyen et al. [14] proposed a highly integrated hybrid model that combines ARIMA, Attention Convolutional Neural Networks (ACNN), Quantum Particle Swarm Optimization (QPSO)-LSTM, and XGBoost. This model uses ARIMA to fit the linear components in historical AQI time series, ACNN to extract deep spatiotemporal features from inputs containing pollutant and meteorological variables, QPSO to optimize LSTM hyperparameters to capture temporal dependencies, and finally XGBoost to fine-tune all outputs. Dey et al. [30] proposed a hybrid model, C^2^SLSTM, that combines a one-dimensional convolutional neural network (1D-CNN) with a customized stacked long short-term memory network (CSLSTM). The model takes multivariate pollutant time-series data as input, extracts spatial features via the CNN, and utilizes a stacked LSTM structure to capture long-term temporal dependencies. In the TMSSICX model, Wu et al. [8] combined an improved Catboost with SOABiLSTM in parallel to predict the decomposed high- and low-frequency components, respectively, with the final output generated via XGBoost ensemble. The input features encompassed pollutant, meteorological, and temporal information. Furthermore, regarding multi-model fusion strategies, existing ensemble methods can be categorized into four types: first, fixed-weight fusion, such as Cui et al. [31] using a fixed distance metric to construct an adjacency matrix, and Ordenshiya et al. [32] employing fixed fuzzy rules and clustering thresholds; second, sequential ensemble with residual correction, such as Sreenivasulu et al. [20] cascading CNN-LSTM-MHA with GRU and fusing them via residual correction; third, data-driven ensemble methods, such as Wu et al. [8] using XGBoost to integrate the predicted values of two components, Elabd et al. [33] employing logistic regression as a meta-learner for stacking, Nguyen et al. [14] using XGBoost as a fine-tuning tool, and Chang et al. [34] integrating multiple prediction models based on Pearson correlation coefficients and stacking strategies; Fourth, optimization algorithm-assisted ensemble methods, such as Dai et al. [35] who employed the Improved Particle Swarm Optimization (IPSO) algorithm to optimize a combined LightGBM and XGBoost model, and Duan et al. [36] who fused ARIMA and CNN-LSTM and used the Dung Beetle Optimizer for hyperparameter tuning.

Although the aforementioned hybrid models have achieved significant improvements in accuracy, existing research still has limitations in terms of feature fusion mechanisms. First, regarding the depth of fusion between temporal-dynamic and static features, most studies either employ unidirectional assistance (where static models are used only for front-end filtering or back-end fine-tuning [29]) or remain at the feature-mixing level (directly concatenating static variables with temporal data and inputting them into a single model [9,28]), and have not yet established a systematic architecture that integrates parallel specialized modeling with deep nonlinear integration at the feature level. Second, regarding strategies for modeling seasonal variations, most models still address seasonal heterogeneity only superficially: MD-STGNN-EN [31] relies on a fixed-distance metric and cannot adapt to the dynamic changes in pollutant dispersion characteristics across seasons; the Fuzzy Inference System [32] uses fixed rules to adapt to different pollution types, essentially applying static rules to a dynamic environment; EEMD-GRU [9] selects only the first three IMFs, potentially losing long-term seasonal trend information; and a large number of studies either cover only short-term data [37,38] or simply encode “season” as a categorical feature for input [8,18], lacking systematic attempts to establish independent submodels for spring, summer, autumn, and winter, respectively, so that each submodel can specifically learn the physicochemical patterns of pollution during that season. Finally, regarding fusion strategies, existing ensemble methods generally suffer from a lack of diversity in fusion methods and the absence of learnable mechanisms. For example, while Stacking ensemble [8,34] achieves data-driven nonlinear combinations, its inputs are mostly scalar predictions or low-dimensional features, resulting in significant information loss; The Stacking ensemble model [33], which uses logistic regression as its meta-learner, has fusion capabilities limited by linear relationships; models assisted by optimization algorithms [35,36,39] focus on hyperparameter tuning but do not alter the linear or fixed structure of the fusion layer itself. Therefore, existing research lacks a nonlinear ensemble design that combines deep features from the time-series branch with prediction values from the static branch as joint inputs to a learnable model, while incorporating contextual information such as seasonality to guide the fusion process.

To address the above issues, this study innovatively proposes a dual-branch parallel architecture, aiming to construct an AQI prediction model that can capture short-term dynamic changes while adapting to long-term seasonal patterns and demonstrating stronger generalization capabilities. Regarding input features, this model clearly distinguishes and utilizes two types of information: first, historical time-series features of pollutants and meteorological variables constructed from sliding windows for the time-series branch; second, contextual features for the static branch, comprising basic static features (such as pollutant concentration and hour), cross-combined features, and seasonal labels. By training independent XGBoost for different seasons and employing a learnable nonlinear fusion layer to integrate high-dimensional temporal features with static prediction values, the model aims to systematically address the two key challenges of insufficient information fusion and weak seasonal generalization.

## 3. Materials and Methods

**Figure 1:**
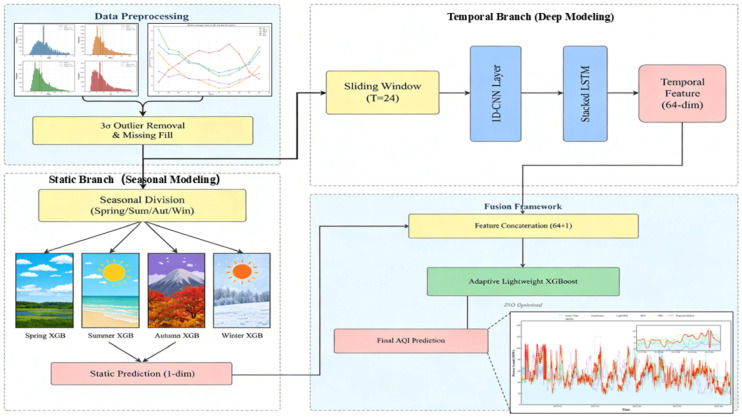
Schematic diagram of the dual-branch parallel hybrid forecasting architecture (CL-XGB-Season).

This study proposes a novel hybrid forecasting architecture (CL-XGB-Season), as illustrated in Figure 1. It aims to enhance the accuracy and generalization capability of AQI prediction by integrating temporal dynamic and static features while adapting to seasonal patterns. The core architecture comprises two specialized branches and a learnable fusion layer: The temporal dynamic branch employs a CNN-LSTM network. It first extracts local temporal patterns through convolutional layers, then utilizes long short-term memory units to capture long-term dependencies, thereby accurately modeling the continuous variation characteristics of AQI. The static feature branch innovatively employs a season-specific XGBoost modeling strategy, training independent XGBoost models for spring, summer, autumn, and winter to learn the distinct influence mechanisms of pollutants and meteorological factors on AQI across different seasons. Finally, the fully trained lightweight XGBoost regression model nonlinearly integrates outputs from both branches, dynamically learning optimal combination weights for temporal and static features across scenarios while incorporating seasonal labels as fusion guides. Particle Swarm Optimization (PSO) algorithms are employed for hyperparameter tuning of critical modules, ensuring optimal performance across all components. This architecture systematically addresses two key challenges: insufficient information fusion and weak seasonal generalization capabilities.

### 3.1. Data Sources and Screening

The experimental data for this study were sourced from the State-controlled Ambient Air Quality Monitoring Station in Chongqing, China, and the China Meteorological Administration. Historical observational data spanning June 2020 to July 2025 were selected to construct the experimental dataset. The dataset features a temporal resolution of one hour, acquired through continuous sampling mode, ensuring the integrity and consistency of the time series.

The dataset contains two types of core parameters: the air quality index (AQI) is used as the prediction target variable to reflect the comprehensive status of air quality; The concentration of key pollutants and meteorological factors were used as the input characteristics of the model. Referring to the research in the field of environmental science, the concentrations of four main pollutants, PM_2.5_, PM_10_, NO_2_ and O_3_, were selected as the core pollution factors. For meteorological data, two factors that have a significant impact on pollutant diffusion and transformation are selected, namely temperature (°C) and precipitation (mm).

To ensure the quality of model inputs, the raw data undergoes preprocessing: random missing values are imputed using the one-hour-ahead and one-hour-behind mean method; outliers are identified and corrected using the 3σ rule; finally, all continuous numerical features are Z-score standardized to eliminate dimensional effects.

To ensure the validity of time series forecasting and validate the model’s seasonal generalization capability, this study employs a hybrid partitioning strategy combining time-sequence and seasonally stratified sampling. The specific approach is as follows:

Test Set: Data from 1 January 2025 to 4 July 2025, used for the final evaluation of the model’s generalization performance. This time period is completely independent of the training and validation sets, effectively testing the model’s ability to forecast future unknown data.

Validation Set and Training Set: These are derived from historical data spanning 1 June 2020 to 31 December 2024. To construct a validation set that matches the training set in terms of seasonal distribution while remaining temporally independent, a stratified sampling method was employed: 30 days of complete daily data (24 samples per day) were randomly selected from each season of every year to form the validation set, totaling 570 samples. It should be noted that this sampling process is conducted independently within each season of every year, and the sampled data does not overlap temporally with the training set. After sampling, these samples were removed from the original dataset, and all remaining historical data constituted the training set. Therefore, the validation set and the training set are completely independent in time, eliminating the risk of data leakage. Since the data begins in June 2020 and the spring data for that year is missing, no samples were drawn from the spring of 2020.

### 3.2. Descriptive Statistical Analysis

To study the overall characteristics and distribution patterns of the data, descriptive statistical analysis was conducted on the entire dataset.

Table 1 presents descriptive statistical analysis of the overall data. Overall, the mean AQI was 47.2 with a median of 45, indicating generally good air quality. However, the standard deviation reached 20.4, reflecting significant fluctuations in air quality. The mean concentrations of PM_2.5_ and PM_10_ were 32.2 μg/m^3^ and 50.3 μg/m^3^, respectively, both exceeding the national ambient air quality secondary standard limits. This highlights the dominant role of particulate matter pollution during the study period and within the study area. The skewness of all pollutant concentration variables exceeded 0.5 (PM_2.5_ skewness reached 1.31), exhibiting a pronounced right-skewed distribution. This confirms the presence of frequent high-pollution event observations in the data, consistent with the sudden and cumulative characteristics of pollution processes.

**Figure 2:**
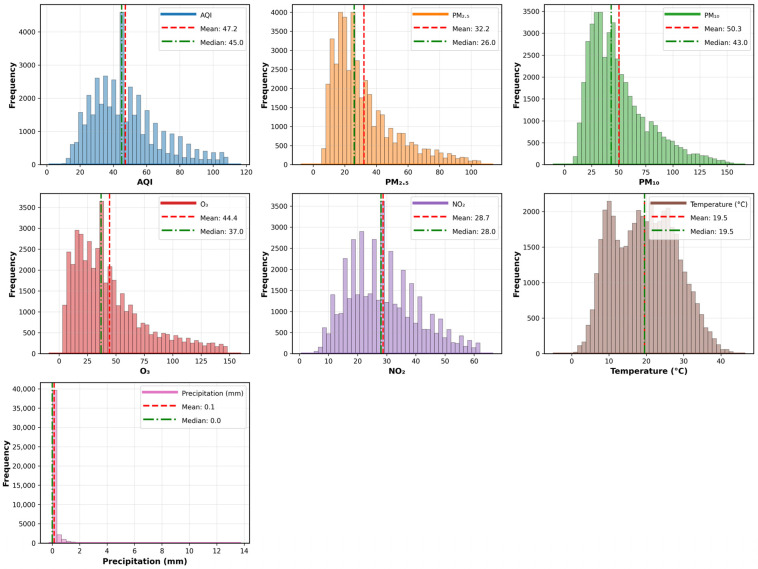
Distribution histograms of core variables (AQI, PM_2.5_, PM_10_, O_3_, NO_2_, Temperature, Precipitation).

Figure 2 shows that PM_2.5_ and PM_10_ exhibit typical right-skewed distributions, with peaks concentrated in the low concentration range and noticeable tails extending into the high concentration range. The Air Quality Index (AQI) follows a slightly right-skewed, near-normal distribution, with its peak centered in the 40–50 range. Temperature follows an approximately normal distribution, with its peak concentrated in the 15–25 °C range. It spans a broad temperature range from 15.6 °C to 34.8 °C, consistent with the seasonal temperature variations typical of a temperate climate. Precipitation exhibits an extremely right-skewed distribution, reflecting the dataset’s predominance of days with no precipitation or light rain.

**Figure 3:**
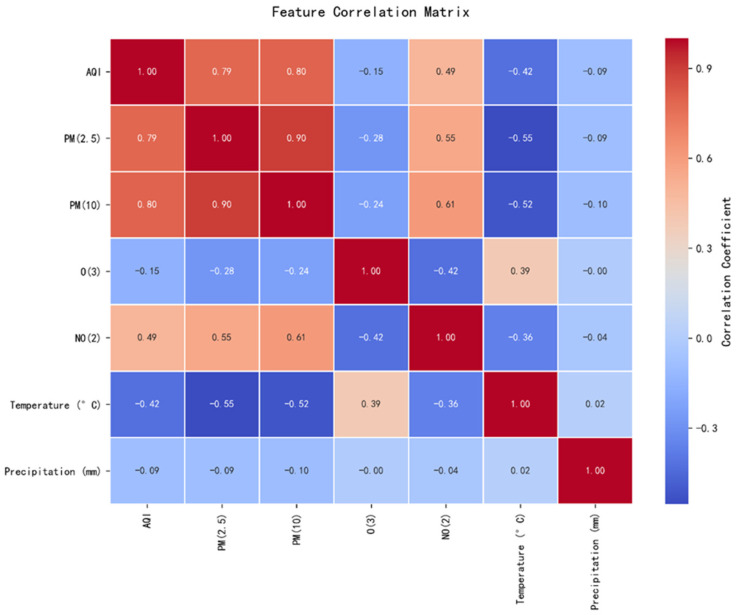
Feature correlation matrix of core variables.

The correlation heatmap results shown in Figure 3 indicate that the Pearson correlation coefficient between AQI and PM_2.5_ reached 0.79, while that with PM_10_ was 0.80, both demonstrating strong positive correlations. In contrast, AQI exhibits weaker correlations with other indicators: moderate correlations with NO_2_ (0.49) and air temperature (−0.42), and weak correlations with O_3_ (−0.15) and precipitation (−0.09). Furthermore, PM_2.5_ and PM_10_ exhibited a high correlation coefficient of 0.90, indicating a significant association. Conversely, O_3_ and NO_2_ showed a moderate negative correlation with a coefficient of −0.42.

This outcome is primarily influenced by pollutant characteristics and environmental mechanisms. As the core factor affecting regional air quality, particulate matter exhibits highly consistent emission sources, with its concentration accumulation directly driving AQI variations. In contrast, the formation and dispersion mechanisms of gaseous pollutants are relatively complex, and their overall emission intensity is lower than that of particulate matter, resulting in a limited contribution to AQI. Among meteorological factors, air temperature exerts differential effects on various pollutant types by influencing photochemical reactions and atmospheric convection. Precipitation primarily removes particulate matter through wet deposition, with negligible effects on gaseous pollutants.

### 3.3. Seasonal Analysis

**Figure 4:**
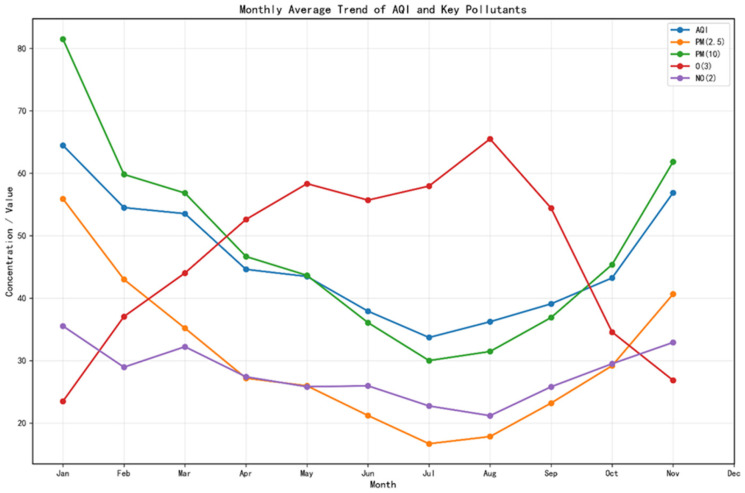
Monthly variations in AQI and major air pollutant concentrations.

Figure 4 reveals a significant seasonal synergy between the influencing factors and the AQI, with winter AQI, PM_2.5_, and PM_10_ concentrations peaking simultaneously. During this period, meteorological conditions—such as low temperatures, low precipitation, and a low boundary layer—hinder the dispersion of pollutants. Concurrently, increased anthropogenic emissions from heating and other sources lead to the accumulation of particulate matter concentrations, becoming the primary driver of rising AQI levels. O_3_ concentrations rise significantly in summer, closely aligning with meteorological conditions characterized by high temperatures and intense solar radiation. This phenomenon can be attributed to the formation mechanism of photochemical pollution: under conditions of intense solar radiation and high temperatures, nitrogen oxides (NO_x_) and volatile organic compounds (VOCs) generated by vehicle exhaust and industrial emissions undergo a series of complex photochemical reactions in the atmosphere, producing secondary pollutants primarily consisting of ozone (O_3_). This reaction process peaks during the afternoon and early evening, leading to a significant rise in O_3_ concentrations during this period, which in turn exerts a phased impact on the AQI. At the same time, frequent summer precipitation and enhanced atmospheric convection facilitate the wet deposition and dispersion of particulate matter, causing concentrations of primary pollutants such as PM_2.5_ and PM_10_, as well as the overall AQI, to drop to their lowest levels of the year. During the transitional seasons of spring and autumn, pollutant concentrations fall between those of winter and summer, exhibiting characteristics of mixed pollution. In early spring, PM_10_ levels tend to fluctuate due to the influence of dust transport, while autumn exhibits transitional pollution characteristics typical of both summer and winter.

**Figure 5:**
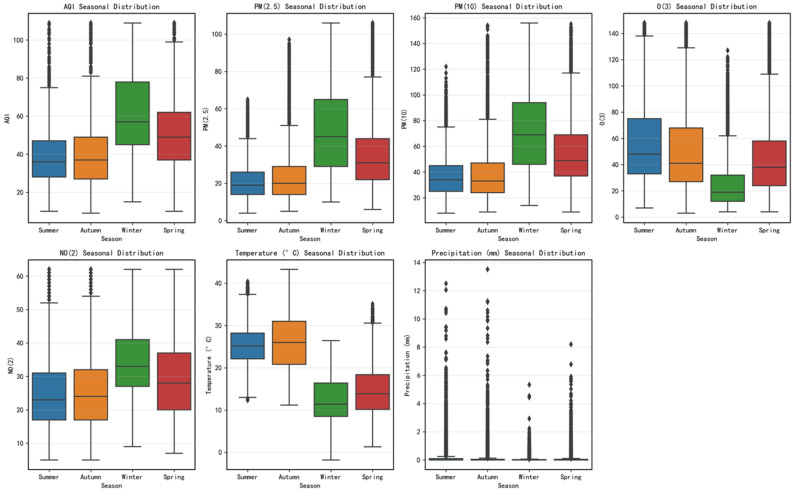
Seasonal distribution boxplots of AQI, key pollutants, and meteorological factors. The black dots in each boxplot indicate outliers (extreme values), and different colors represent different seasons: blue for Summer, orange for Autumn, green for Winter, red for Spring.

Figure 5 further quantifies the differences in seasonal data distribution. Winter not only exhibits the highest median AQI but also the largest interquartile range (IQR). In contrast, summer shows the most concentrated AQI distribution, reaching the lowest level throughout the year. From a pollutant perspective, PM_2.5_ distribution in winter exhibits a pronounced right skew and high outlier values, confirming the frequent occurrence of heavy pollution episodes. Conversely, O_3_ distribution spans the widest range in summer, reflecting the instability of photochemical pollution processes. Spring and autumn pollutant distributions fall between winter and summer extremes, representing transitional mixed pollution patterns. These seasonal variations in distribution patterns necessitate that prediction models possess sufficient adaptability to accommodate data heterogeneity across different seasons.

## 4. Model Construction

### 4.1. Feature Engineering Based on a Dual-Branch Architecture

This feature engineering approach incorporates multiple targeted techniques, including: (i) sliding window sequence extraction (SEQ), (ii) basic static feature extraction (BASIC), (iii) pollutant-meteorological factor cross-combination (CROSS), and (iv) seasonal label mapping (SEASON). These techniques address core pollution forecasting requirements by extracting features at key temporal scales and scenarios: the sliding window is fixed at 24 h to precisely capture intraday temporal dependencies of AQI; basic static features and cross-features cover intrinsic correlations between pollutants and between pollutants and meteorological conditions; and seasonal labels provide a classification basis for seasonal modeling. Ultimately, the initial six categories of raw features are expanded into a multidimensional feature set tailored for the dual-branch model. Meanwhile, the raw data involved in this study comprises three types of heterogeneous information: time-series data (hourly observations of AQI, pollutants, and air temperature), static numerical data (current pollutant concentrations and meteorological factors), and static categorical data (hour codes and seasonal labels). The differing properties of these three data types dictate a divergent processing design: time-series data retains its temporal structure via a sliding window (SEQ) and is converted into a three-dimensional tensor, enabling the CNN-LSTM branch to extract dynamic evolution patterns; static data is organized into a two-dimensional feature matrix through basic feature extraction (BASIC) and cross-feature construction (CROSS), allowing the XGBoost branch to fit nonlinear static patterns; Seasonal labels (SEASON) further serve as grouping identifiers for data partitioning in seasonal modeling. Each processing path operates independently, ensuring that each data category enters the corresponding module in the most suitable form, thereby avoiding the issues of feature competition and information confusion that may arise from feeding heterogeneous data into a single model.

Based on the core logic and data characteristics of AQI forecasting, the construction of time-series features uses the previous time step (t − 1) as the endpoint, establishing a 24 h sliding window that covers the historical observation sequence within the [t − 24, t − 1] time interval. Within this window, core variables highly correlated with AQI are selected, specifically including AQI, PM_2.5_, PM_10_, NO_2_, O_3_, and air temperature, for a total of six indicators. Each feature undergoes Z-score normalization within its respective sliding window to eliminate dimensional differences and enhance model training convergence efficiency. This process transforms each sample point into a three-dimensional tensor with dimensions (1, 24, 6). The entire training set thus forms a feature tensor with explicit short-term dependencies for the temporal branch input. The sliding window mechanism inherently introduces lagged features, enabling the model to directly learn how past states within the preceding 24 h influence future outcomes. The sliding window step size is set to 1 h, meaning the model employs a single-step forecasting strategy, using the 24 h history within the window to predict the AQI value for the next hour.

At the static feature level, foundational static features are first extracted directly, including core pollutant concentrations (PM_10_, NO_2_), temporal dimension information (hour), and seasonal identifiers (1 = Spring/2 = Summer/3 = Autumn/4 = Winter). These features form the foundational skeleton of static patterns. Cross-combination features (CROSS) are further constructed, including two key cross-terms: “PM_2.5_/PM_10_ ratio” and “temperature × O_3_.” Among these, the “PM_2.5_ to PM_10_ ratio” reflects the composition characteristics of particulate matter pollution and serves as a critical indicator during winter when fine particulate matter dominates pollution. The design of the “temperature × O_3_” cross-feature stems from the positive correlation between ozone formation rates and temperature; under high-temperature conditions, photochemical reactions accelerate, leading to a rapid increase in O_3_ concentrations. By constructing this interaction term, the model can capture the synergistic effect between temperature and O_3_, thereby more effectively learning the nonlinear response patterns dominated by photochemical pollution during high-temperature periods in summer and enhancing the static branch’s ability to fit complex pollution scenarios.

Given the pronounced seasonal variations in AQI patterns—heavy pollution in winter, ozone dominance in summer, and significant PM_10_ fluctuations in spring—the Season Label Mapping technique (SEASON) was employed. This approach decomposed the “season” column into four categorical labels (1–4), providing explicit guidance for seasonal XGBoost modeling. This enables the model to learn season-specific patterns while avoiding generalization degradation caused by cross-seasonal feature interference.

Feature engineering strictly avoids data leakage risks and aligns with feature attributes. The target variable AQI undergoes no additional transformation, as scaling adjustments in time series forecasting distort intrinsic evolution patterns. Moreover, transformations relying on future data risk data leakage, compromising model reliability. Season labels, treated as categorical features, undergo only mapping processing. They are excluded from time-based operations like time-series shifting or numerical cross-folding to preserve the validity of their categorical attributes.

To achieve deep complementarity between temporal and static features, a fusion adaptation process is performed in the final stage of feature engineering. The 64-dimensional temporal feature vector from the CNN-LSTM branch is concatenated with the 1-dimensional static prediction value from the seasonally split XGBoost branch, constructing a 65-dimensional fusion feature matrix. The dimensional design of the fused features preserves the dynamic information integrity of the time-series features while incorporating the long-term patterns and seasonal variation information from the static features. Simultaneously, training and adaptation through the lightweight XGBoost regression model ensure the effective fusion of both feature types. This ultimately provides high-quality input features for AQI prediction that combine dynamic responsiveness with seasonal adaptability.

### 4.2. Prediction Model Based on Heterogeneity-Driven Dual-Branch Architecture

This study constructs a forecasting framework that decouples dynamic and static characteristics through seasonal modeling integration. By employing specialized designs for time-series and static branches and introducing a fusion mechanism guided by seasonal labels, the framework enhances the model’s adaptability to seasonal shifts and overall forecasting accuracy. This framework is not a simple model combination but rather decouples complex forecasting tasks into two subproblems with distinct physical significance, establishing a collaborative mechanism between them.

Traditional single models struggle to simultaneously capture both the short-term fluctuations and long-term seasonal patterns of AQI. This challenge stems from the inherent significant heterogeneity within AQI data itself, where driving factors vary across different time scales and dominant mechanisms differ across seasons. This framework employs a decoupled design to construct two functionally specialized prediction pathways:

The dynamic temporal pathway focuses on capturing short-term temporal fluctuations driven by the interaction between meteorological conditions and pollutants. It employs a sliding window to extract local features and utilizes LSTM layers to model long-term dependencies, encoding dynamic changes. This pathway primarily addresses the non-stationary hourly fluctuations in AQI.

The Static Pattern Pathway focuses on fitting long-term static patterns determined by pollutant emissions and seasonal climate backgrounds. It directly utilizes static features such as pollutant concentrations, hours, and seasons, enhancing representational capacity through feature cross-product analysis.

Outputs from both pathways are integrated through a learnable fusion layer. This layer employs a lightweight XGBoost regressor that learns joint contribution weights for dynamic and static features, achieving information complementarity and scenario adaptation rather than simple weighted averaging.

The design of seasonal modeling stems from the seasonal heterogeneity of AQI data: PM10 dominates in spring, O_3_ concentrations exhibit a nonlinear positive correlation with temperature in summer, and PM_2.5_ tends to accumulate in winter, with significant differences in pollution drivers across seasons. A single static model struggles to dynamically adjust feature weights to accommodate this heterogeneity, whereas seasonal submodels learn an optimal parameterized representation for each season’s data distribution. This enables the model to enhance feature responses to core seasonal pollutants. This mechanism effectively improves the model’s generalization ability across seasonal variations, addressing the pain point of poor seasonal adaptability in single models.

**Figure 6:**
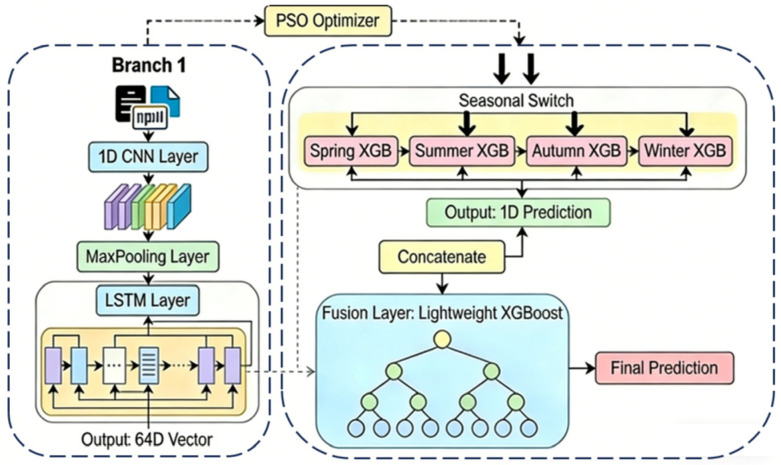
Detailed structure diagram of the dual-branch heterogeneous fusion prediction. model.

The fusion stage concatenates outputs from the temporal and static branches into a trainable XGBoost model, enabling it to autonomously learn optimal feature combinations from the data. The overall architecture is illustrated in Figure 6.

#### 4.2.1. Temporal Branch: CNN-LSTM

CNN-LSTM serves as the core model for temporal feature extraction in this study. As a key component of the dual-branch framework, it is specifically designed to capture both short-term local fluctuations and long-term dependencies within AQI time series, addressing the limitations of traditional single-time-series models that struggle to balance “local feature capture” and “long-term correlation mining.” Through a stacked architecture combining CNN and LSTM, this model first extracts hourly level spatiotemporal patterns of pollutant concentrations and meteorological factors, then uncovers cross-temporal dynamic correlations, ultimately outputting high-dimensional temporal feature vectors. These provide precise short-term dynamic information support for subsequent fusion layers. The computational processes of the CNN and LSTM follow the standard architecture defined in Reference [11].

The one-dimensional convolutional layer employs sliding operations of convolution kernels along the temporal dimension to extract collaborative variation patterns among multiple variables within local time windows. For an input feature sequence $x\in R^{t\times f}$, its convolution operation is defined as:

(1) H[t,k]=ReLU∑i=1F∑s=0S−1W[s,i,k]⋅X[t+s,i]+b[k]

t denotes the sequence length, F represents the feature dimension, s indicates the convolution kernel width, $W\in R^{s\times f\times k}$ and $b\in R^{k}$ are trainable parameters, W is the convolution kernel weight matrix, b is the bias term, X denotes the number of output feature maps, k represents the number of output feature maps, and ReLU is the activation function. Multiple parallel convolutional kernels capture local patterns across different temporal scales.

Subsequently, the pooling layer downsamples features to enhance robustness. The output of this layer is a refined sequence of high-level features.

The Long Short-Term Memory (LSTM) network employs an LSTM layer with 64 hidden units. Through its gating mechanism (forget gate, input gate, output gate), it dynamically adjusts the retention and updating of temporal information, effectively capturing long-term dependencies across time intervals. Given an input feature sequence, the computational process of an LSTM unit at each time step t is as follows:

The candidate cell state integrates information from the current input $X_{t}$ and the previous hidden state $h_{t-1}$ through a nonlinear transformation:

(2) $${\tilde{C}}_{t}=\tanh\left(W_{c}\cdot [h_{t-1},x_{t}]+b_{c}\right)$$

where $W_{c}$ is the weight matrix of candidate states, i.e., trainable parameters; $C_{t}$ is the candidate cell state; $h_{t-1}$ is the LSTM output at time step t − 1; $x_{t}$ is the current input; and $b_{c}$ is the bias vector of candidate states.

The input gate $i_{t}$, forget gate $f_{t}$, and output gate $o_{t}$ generate gating signals through the sigmoid function:

(3) $$i_{t}=\sigma \left(W_{i}\cdot [h_{t-1},x_{t}]+b_{i}\right)$$

(4) $$f_{t}=\sigma \left(W_{f}\cdot [h_{t-1},x_{t}]+b_{f}\right)$$

(5) $$o_{t}=\sigma \left(W_{o}\cdot [h_{t-1},x_{t}]+b_{o}\right)$$

where $W_{i}$, $W_{f}$ and $W_{o}$ denote the weight matrices for the input gate, forget gate, and output gate, respectively; $b_{i}$, $b_{f}$ and $b_{o}$ denote the bias vectors for the input gate, forget gate, and output gate, respectively.

The cell state $C_{t}$ is updated based on the gate signals:

(6) $$C_{t}=f_{t}⊙C_{t-1}+i_{t}⊙{\tilde{C}}_{t}$$

The hidden state $h_{T}$ as the unit output:

(7) $$h_{T}=o_{t}⊙\tanh\left(C_{t}\right)$$

Finally, the hidden state $h_{T}$ of the last time step in the LSTM is output as the context vector for the entire temporal branch:

(8) $$z_{temp}=Pooling\left(\left[h_{1},h_{2},\ldots ,h_{T}\right]\right)$$

$z_{temp}$ denotes the output vector of the temporal branch, while $h_{1},h_{2},\ldots ,h_{T}$ represent the hidden states at each time step.

#### 4.2.2. Static Splits: Seasonal XGBoost

Static feature branching employs the Extreme Gradient Boosting (XGBoost) algorithm to model static and cross-features processed through feature engineering. XGBoost is an ensemble learning algorithm based on Gradient Boosting Decision Trees (GBDT). Its core principle involves sequentially constructing a series of weak learners (decision trees) and combining their predictions in a stacked manner to form a strong learner. The mathematical principles of the XGBoost algorithm are based on the gradient-boosting framework proposed in [14,29].

XGBoost trains the model by minimizing an objective function comprising a loss function and a regularization term, defined as follows:

(9) $$L\left(\Theta \right)=\sum_{i=1}^{n}l\left(y_{i},{\hat{y}}_{i}\right)+\sum_{k=1}^{K}\Omega \left(f_{k}\right)$$

Among these, $l\left(y_{i},{\hat{y}}_{i}\right)$ is the loss function measuring the discrepancy between the actual value $y_{i}$ and the predicted value ${\hat{y}}_{i}$. L represents the objective function, while $\Omega \left(f_{k}\right)$ is the regularization term used to control the complexity of the kth tree, expressed as:

(10) $$\Omega \left(f_{k}\right)=\gamma T+\frac{1}{2}\lambda \sum_{j=1}^{T}w_{j}^{2}$$

T denotes the number of leaf nodes in the tree, $w_{j}$ represents the weight of leaf node j, and γ and λ are regularization parameters used to penalize model complexity and prevent overfitting.

The training process employs a forward stepwise algorithm with gradient boosting. At iteration t, the model fits a new decision tree $f_{t}$ based on the prediction residuals of the current ensemble model $F_{t-1}\left(x\right)$:

(11) $$r_{i}^{\left(t\right)}=-{\left[\frac{𝜕l\left(y_{i},{\hat{y}}_{i}\right)}{𝜕{\hat{y}}_{i}}\right]}_{{\hat{y}}_{i}=F_{t-1}\left(x_{i}\right)}$$

where $r_{i}^{\left(t\right)}$ denotes the pseudo residual for the i-th sample in iteration t; $\frac{𝜕l}{𝜕{\hat{y}}_{i}}$ denotes the partial derivative of the loss function with respect to the predicted value; $x_{i}$ is the feature vector of the i-th sample. The loss function is approximated using a second-order Taylor expansion, and the optimal tree structure parameters are sought to generate the new tree in a greedy manner:

(12) $$L^{\left(t\right)}\approx \sum_{i=1}^{n}\left[l\left(y_{i},F_{t-1}\left(x_{i}\right)\right)+g_{i}f_{t}\left(x_{i}\right)+\frac{1}{2}h_{i}f_{t}^{2}\left(x_{i}\right)\right]+\Omega \left(f_{t}\right)$$

where $g_{i}$ $h_{i}$ represent the first-order and second-order gradients of the loss function, respectively. The final model’s prediction is the sum of the predictions from all K trees:

(13) $${\hat{y}}_{i}=\sum_{k=1}^{K}f_{k}\left(x_{i}\right)$$

Given the seasonal variation in AQI formation mechanisms, this study proposes a seasonal XGBoost modeling strategy. Heterogeneous global training data is divided into four homogeneous subsets based on seasonal labels: spring, summer, autumn, and winter (1 = spring, 2 = summer, 3 = autumn, 4 = winter). For each seasonal dataset, one XGBoost submodel is trained. The objective function for each seasonal submodel $M_{s}$ (s = 1, 2, 3, 4) can be formally expressed as:

(14) $$L_{s}\Theta =\sum_{i\in D_{s}}l\left(y_{i},{\hat{y}}_{i}\right)+\sum_{k=1}^{K_{s}}\Omega \left(f_{k}^{\left(s\right)}\right)$$

$D_{s}$ denotes the set of training samples belonging to season s. $L_{s}$ represents the objective function of the submodel for season s; $f_{k}^{\left(s\right)}$ denotes the k-th tree of the submodel for season s.

Simultaneously employing globally optimized parameters via PSO, the submodels enhance their adaptability to corresponding seasonal characteristics. During the forecasting phase, the system invokes the submodel for the input data’s designated season based on its seasonal label, generating a one-dimensional static forecast value to ensure alignment with seasonal patterns.

#### 4.2.3. Fusion Layer: Capable of Learning Nonlinear Fusion

The design of this fusion module stems from the fundamental differences in the output information of the dual branches. The fusion layer employs an XGBoost regressor to perform nonlinear mapping on the concatenated features. Its objective function follows the gradient boosting framework proposed in [14,29]. The temporal branch outputs a high-dimensional contextual feature vector $z_{t}$, which encodes the local fluctuation patterns of pollutant concentrations and meteorological variables on an hourly scale, as well as the long-term dependencies across time periods. The static branch (seasonal XGBoost) takes the static features described in Section 4.1 as input and outputs a scalar prediction value $s_{t}$ based on seasonal patterns. These two types of features differ fundamentally in both physical meaning and data form: $z_{t}$ is a high-dimensional continuous vector containing rich temporal dynamic patterns, while $s_{t}$ is a scalar that encapsulates static seasonal patterns.

To achieve this integration, this study employs a fusion strategy combining feature concatenation with nonlinear regression. First, the 64-dimensional feature vector output by the time-series branch is concatenated with the 1-dimensional prediction value output by the static branch to form a 65-dimensional joint feature vector:

(15) $$F_{t}=[z_{t};s_{t}]\in R^{D+1}$$

Specifically, let the output of the temporal dynamic branch (CNN-LSTM) be a high-dimensional feature vector $z_{t}\in R^{D}$, and let the output of the seasonal XGBoost model in the static feature branch be a scalar preliminary forecast $s_{t}\in R$.

Subsequently, a gradient-boosted decision tree model is employed as the fusion model $g_{fuse}$, whose objective is to fit a nonlinear function from the joint features $F_{t}$ to the true target $y_{t}$, trained under a minimum regularization objective. The rationale for selecting XGBoost as the fusion model is as follows: (1) tree structure inherently supports the fitting of nonlinear relationships and can capture the complex interactions between $z_{t}$ and $s_{t}$; (2) the gradient boosting strategy, prediction residuals can be progressively corrected to improve fusion accuracy; (3) to neural networks, XGBoost exhibits greater robustness and interpretability when fusing a small number of features. The objective function of the fusion model is as follows:

(16) $$L=\sum_{i}l\left(y_{i},{\hat{y}}_{i}\right)+\sum_{k}\Omega \left(f_{k}\right)$$

(17) $${\hat{y}}_{i}=g_{fuse}\left(F_{i}\right)=\sum_{k=1}^{K}f_{k}\left(F_{i}\right)$$

$f_{k}$ denotes a single regression tree, K represents the total number of trees, and $\Omega \left(f_{k}\right)$ serves as the regularization term controlling tree complexity. This model progressively refines prediction residuals through additive modeling and gradient boosting.

The learnability of this fusion mechanism is reflected in the fact that the fusion model does not use fixed weights; instead, it dynamically learns the relative importance of $z_{t}$ and $s_{t}$ for each sample through the splitting rules within its tree structure. Mathematically, XGBoost gradually approximates the objective function through the additive model $g_{fuse}$. Each new tree $f_{k}$ is generated based on the negative gradient direction of the current residual, allowing the fusion weights to adaptively adjust according to the feature distribution of the input samples. During forward inference, the model automatically determines, based on specific input values, the extent to which it should rely on temporal contextual features or static patterns for prediction. During the prediction phase, all input variables are assumed to be available without delay at the time of prediction. This assumption aligns with the hourly data release pattern of air quality monitoring systems and is suitable for real-world early warning scenarios.

## 5. Experimental Verification and Analysis

### 5.1. Evaluation Indicators

To quantitatively evaluate and compare the predictive performance of different models, this study employs root mean square error (RMSE), mean absolute error (MAE), mean absolute percentage error (MAPE), and coefficient of determination (R^2^) as core evaluation metrics. RMSE is more sensitive to large errors and effectively reflects the overall deviation between predicted and actual values, serving to evaluate model prediction stability. MAE reflects the average absolute deviation, yielding intuitive and easily interpretable results that directly indicate the model’s average prediction error level. MAPE presents the average error as a percentage, facilitating comparisons of predictive performance across different pollution concentration ranges and seasons. R^2^ measures the model’s ability to explain the variance of the target variable, with values ranging from [0, 1]. A value closer to 1 indicates better model fitting to the data patterns. The calculation formulas are as follows:

(18) $$RMSE=\sqrt{\frac{1}{N}\sum_{i=1}^{N}{\left(y_{i}-{\hat{y}}_{i}\right)}^{2}}$$

(19) $$MAE=\frac{1}{N}\sum_{i=1}^{N}\left|y_{i}-{\hat{y}}_{i}\right|$$

(20) $$R^{2}=1-\frac{\sum_{i=1}^{N}{\left(y_{i}-{\hat{y}}_{i}\right)}^{2}}{\sum_{i=1}^{N}{\left(y_{i}-\bar{y}\right)}^{2}}$$

(21) $$MAPE=\frac{1}{n}\sum_{i=1}^{n}\frac{\left|y_{i}-{\hat{y}}_{i}\right|}{y_{i}}\times 100$$

$y_{i}$ represents the true value,${\hat{y}}_{i}$ denotes the predicted value, ${\bar{y}}_{i}$ signifies the mean of the true values, and N indicates the total number of test samples. Through these multidimensional metrics, a comprehensive evaluation of the model’s predictive accuracy, robustness, and goodness-of-fit can be conducted.

### 5.2. Feature Importance Analysis and Validation

**Figure 7:**
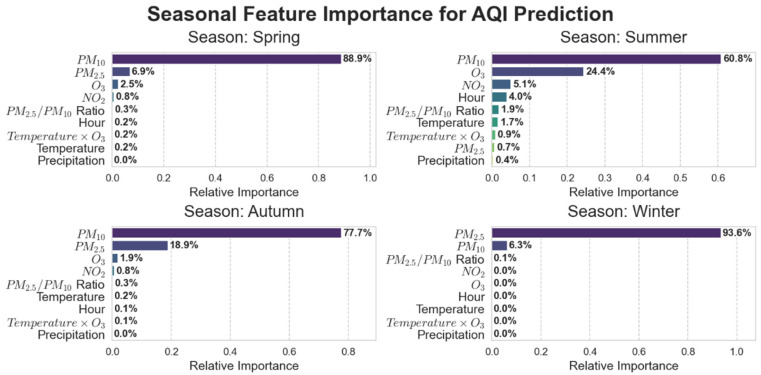
Seasonal feature importance scores from the XGBoost model.

**Figure 8:**
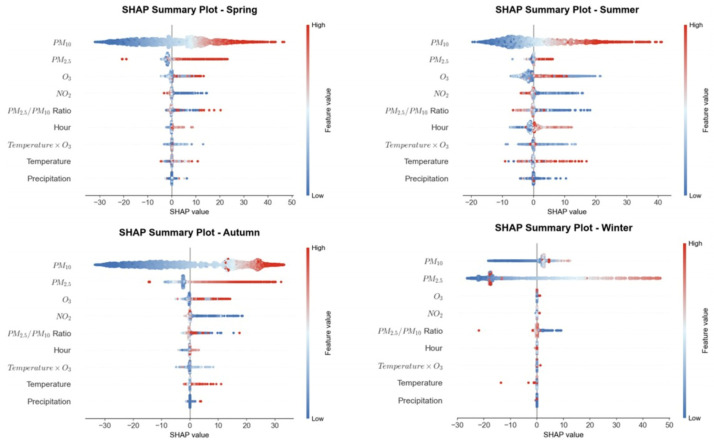
SHAP summary plots of feature impacts on model output by season.

**Figure 9:**
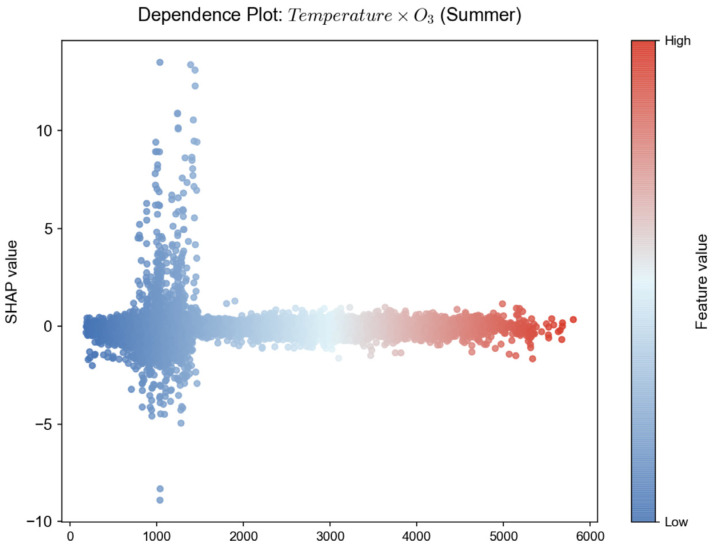
Dependence plot of the interaction term “Temperature × O_3_” and its impact on AQI in summer.

Feature selection based on domain knowledge retains core pollutant characteristics such as PM_2.5_, PM_10_, NO_2_, and O_3_ from raw monitoring data. The AQI prediction model must adapt to pollution patterns across different seasons and weather conditions, with meteorological factors serving as the core enabler for the model’s “cross-scenario generalization.” Ref. [14]’s research indicates that Seoul’s topography (surrounded by mountains and ocean) causes pollutants to linger, essentially reflecting how terrain indirectly influences AQI by affecting local meteorological conditions. Rabie et al.’s CNN-Bi-LSTM model exhibits reduced accuracy when predicting sudden pollution spikes, partly due to insufficient integration of real-time meteorological data. Based on this, we selected key meteorological features such as temperature and precipitation, along with temporal features like “hour” and “season.” During the refinement phase, a data-driven approach optimized the feature set. Features with Pearson correlation coefficients > 0.3 were retained, as shown in Figure 3. PM_2.5_ (0.79) and PM_10_ (0.8) exhibit strong positive correlations with AQI, while temperature (−0.41) shows a moderate correlation. No significant redundancy exists among core features. Furthermore, the “temperature × O_3_” feature was constructed to quantify the synergistic effect of high temperatures on O_3_ formation. As shown in the feature importance analysis in Figure 7, although this cross-feature has a low global contribution, its effect exhibits significant seasonal specificity, primarily manifesting in summer pollution scenarios. Further analysis of the seasonal distribution of SHAP values (Figure 8) reveals that when Air Temperature × O_3_ > 3000, the SHAP values for this feature are predominantly positive, and such high-value samples are highly concentrated in the summer. Furthermore, the relationship curve between “Air Temperature × O_3_” and AQI (Figure 9) shows a significant positive nonlinear correlation between the two during the summer period. From an atmospheric physicochemical perspective, under high-temperature and strong solar radiation conditions in summer, nitrogen oxides and volatile organic compounds in the atmosphere undergo photochemical reactions to form ozone, with this process peaking from the afternoon through the evening. The positive nonlinear correlation captured by the model through the “Temperature × O_3_” interaction feature is consistent with the aforementioned photochemical patterns, indicating that this feature can effectively identify prediction scenarios dominated by summer photochemical pollution.

**Figure 10:**
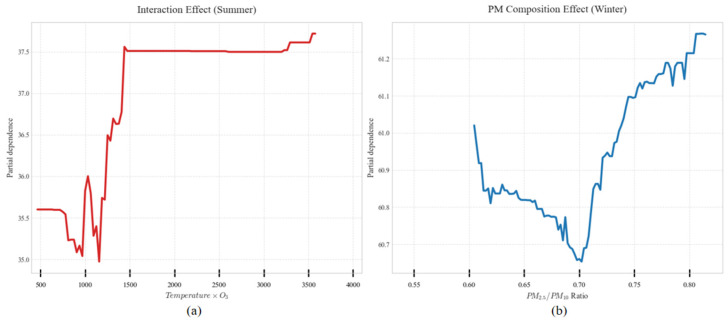
Interaction effect plots: (**a**) Temperature × O_3_ interaction (Summer); (**b**) PM_2.5_/PM_10_ ratio effect (Winter).

Additionally, to address the seasonal characteristics dominated by particulate matter pollution in winter, we introduced the PM_2.5_/PM_10_ ratio to characterize differences in particulate matter composition. The winter bias dependence curve for this ratio, shown in Figure 10, indicates a pronounced trough in the bias effect on AQI when the ratio falls within the 0.65–0.70 range. Beyond 0.70, the bias effect exhibits a rapid upward trend as the ratio increases. This pattern aligns with actual observations showing that pollution worsens in winter due to the increased proportion of fine particulate matter (PM_2.5_), indicating that the model can effectively capture this seasonal characteristic.

### 5.3. Experimental Results and Analysis

The training set was constructed based on the validation set data, with input features comprising the 64-dimensional time-series feature vector from the CNN-LSTM branch and the 1-dimensional static prediction value from the seasonally split XGBoost branch. The target values were the actual observed AQI measurements. As shown in Table 2, the overall nRMSE of the training set is 0.084, with an R^2^ of 0.9930, nMAE of 0.063, and MAPE of 3.30%. indicating the model has effectively learned the temporal fluctuation patterns, static correlation features, and seasonal variation patterns of AQI. By season, winter exhibits the highest nRMSE. Considering winter’s higher pollution concentrations and complex composition, the R^2^ still reaches 0.9919, maintaining excellent fitting performance.

Table 3 shows the validation set’s overall metrics: nRMSE = 0.198, R^2^ = 0.9668, nMAE = 0.088. While slightly higher than the training set, these values remain at a high level, indicating the model exhibits no overfitting and possesses strong generalization capabilities. Among seasonal variations, the R^2^ value for summer was the lowest at 0.8730, while autumn shows nRMSE = 0.246 and R^2^ = 0.9367. Both summer and autumn performance metrics present room for improvement.

The test set covers real-world application data from January to July 2025. Table 4 shows that the base model achieves overall nRMSE = 0.251, R^2^ = 0.9368, nMAE = 0.098, and MAPE = 3.67%, validating its practical applicability. Among the seasons, autumn lacks sufficient data due to coverage ending in July 2025. Spring exhibited the highest RMSE and MAE values, alongside the lowest *R*^2^ value. This primarily stems from the unique characteristics of spring data, including significantly higher average PM10 levels compared to other seasons and substantial precipitation fluctuations.

To further enhance the overall model performance and seasonal balance, PSO-based global parameter tuning was conducted based on the baseline model results. The tuning process used the base model parameters as initial values and performed global optimization on core parameters such as learning rate, batch size, n_estimators, and max_depth for the XGBoost fusion layer. The search ranges for each hyperparameter and the final optimal values selected are summarized in Table 5. The optimal settings were obtained: LSTM unit count = 106, learning rate = 0.00484, XGBoost base parameters n_estimators = 144, fusion_lr = 0.05, xgb_depth = 4; fusion_depth = 5, fusion_estimators = 78.

After parameter tuning, the performance significantly improved. Table 6 shows that the overall nRMSE on the training set is 0.197, R^2^ = 0.9611, and nMAE = 0.063. The fusion model outperforms the SSA-LSTM-XGBoost hybrid model [40] proposed in 2025 (R^2^ = 0.9554). Compared to pure time series models like Transformer, our model achieves over 44% higher prediction accuracy by integrating static features and seasonal modeling. Although slightly more volatile than the baseline model, its seasonal performance is more balanced. The overall nRMSE on the validation set decreased to 0.146, while R^2^ increased to 0.9821. The overall nRMSE on the test set decreased to 0.197, and R^2^ improved to 0.9611. For each season, both nRMSE and nMAE decreased, while R^2^ significantly improved. This demonstrates synergistic optimization of overall accuracy and seasonal balance.

#### Model Prediction Analysis

**Figure 11:**
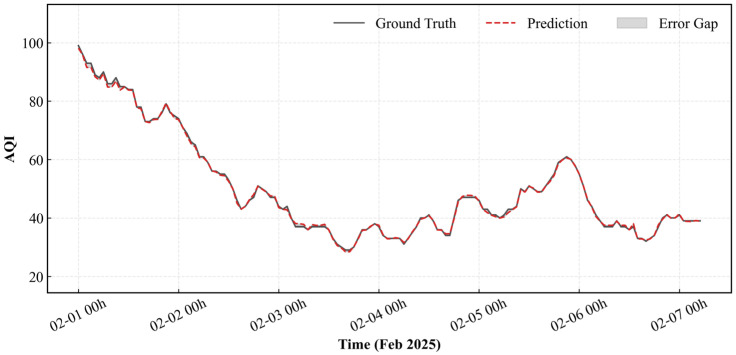
Time series comparison of predicted and actual AQI values (February 2025).

**Figure 12:**
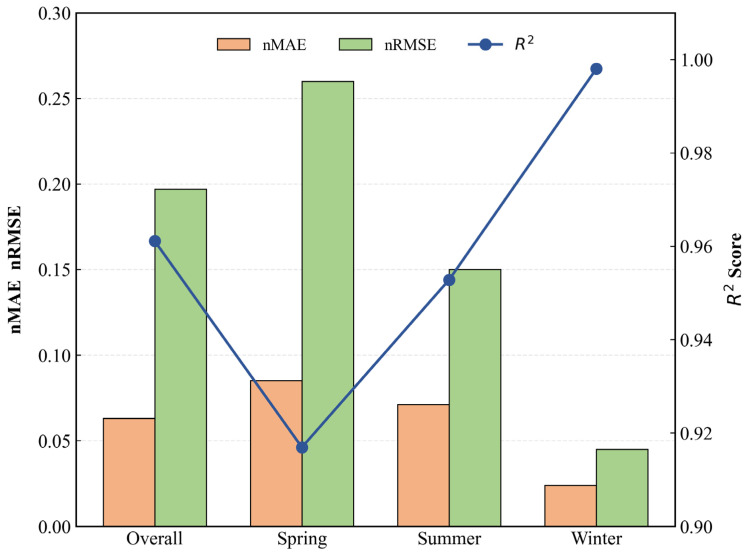
Seasonal performance metrics (nRMSE, nMAE, R^2^) of the proposed model.

The predictive performance of the proposed model was evaluated on the spring, summer, and winter test datasets for 2025, with results visualized in Figure 11 and Figure 12. Throughout the entire summer period, the model accurately captured the trend of AQI fluctuations even during periods of significant volatility, demonstrating its dual capability to track both seasonal patterns and sudden spikes in air quality.

Seasonal performance metrics reveal the model achieved its lowest nRMSE and nMAE in winter, with an R^2^ coefficient reaching 0.998. In spring and summer, the model similarly demonstrated stable performance—nRMSE values of 0.260 and 0.150, respectively, with R^2^ coefficients consistently above 0.9. This indicates the model effectively captures the variance characteristics of AQI data across different seasons.

As shown in Figure 11, the model’s prediction error and deviation from the actual benchmark are kept within a narrow range. In terms of performance metrics, the model achieved its lowest prediction nMAE of 0.024 during the winter, and the fluctuation in error across seasons remained below 0.1. This indicates that the model is capable of tracking the dynamic changes in AQI over time and maintains relatively stable predictive performance across different seasons.

### 5.4. Melting Experiments and Comparative Analysis

To evaluate the core roles of “dual-branch fusion,” “seasonal modeling,” and the “fusion layer,” and to clarify each component’s contribution to model performance, we constructed nine ablation variants. These include: a single CNN-LSTM model, a CNN model without LSTM layers and static features, a dual-branch model without seasonal components, a fusion model guided by non-seasonal features, a fixed-weight fusion model, a direct concatenation fusion model, and a single-model seasonally encoded XGBoost. These eight variants were compared under consistent input and training settings. Detailed results are presented in Table 7, Table 8, Table 9 and Table 10.

#### 5.4.1. Dual-Branch Fusion Framework Validation

**Figure 13:**
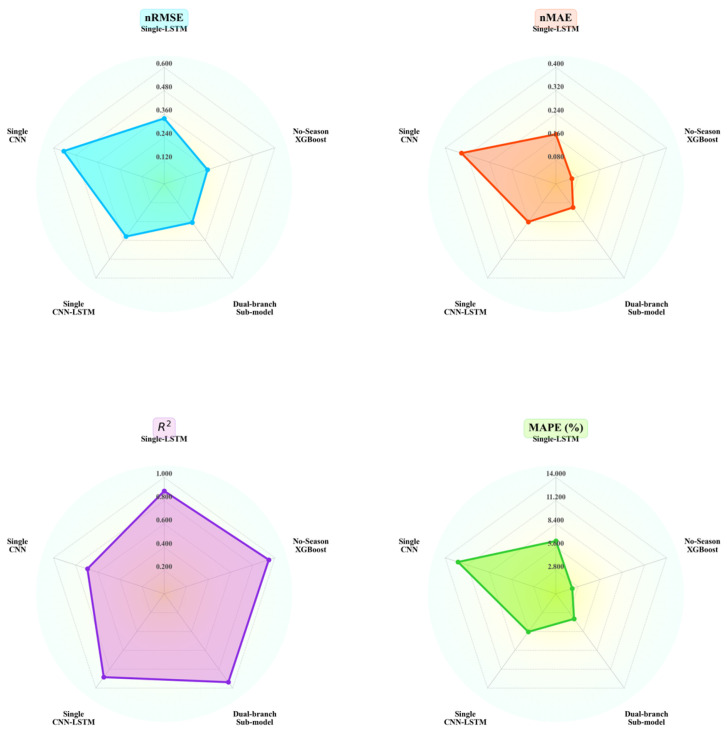
Comprehensive comparison of ablation experiment results across models.

The single LSTM retaining only the temporal branch and the XGboost retaining only the static branch both show an increase in test set nRMSE to over 0.2 in Figure 13 and Table 10, representing an improvement of over 19% compared to the core model. Their R^2^ values both drop below 0.95, indicating that both types of branches contribute equally to modeling accuracy and are indispensable. Conversely, the single CNN variant—which removed both key components of the dual-branch architecture—exhibited the most significant deterioration in performance. Its nRMSE reached 0.545 and R^2^ dropped to 0.6941, representing a 27.78% increase in error compared to the core model. This further confirms that the dual-branch collaborative architecture is fundamental to ensuring high-precision forecasting.

#### 5.4.2. Seasonal Modeling Validation

Given the seasonal dependency of AQI variations and the temporal heterogeneity of pollution-driving mechanisms, the dual-branch model without seasonal components exhibited the most pronounced performance degradation during winter, with an nRMSE reaching 0.143. This closely correlates with the complex characteristics of winter AQI, characterized by high concentrations and strong accumulation. The nRMSE in spring and summer improved by 160.9% and 216.3%, respectively, compared to the core model, further confirming that seasonal modeling effectively avoids the confounding of mechanisms caused by full-sample modeling. The seasonal results for Variant 3 are shown in Table 11.

#### 5.4.3. Fusion Layer Detail Verification

**Figure 14:**
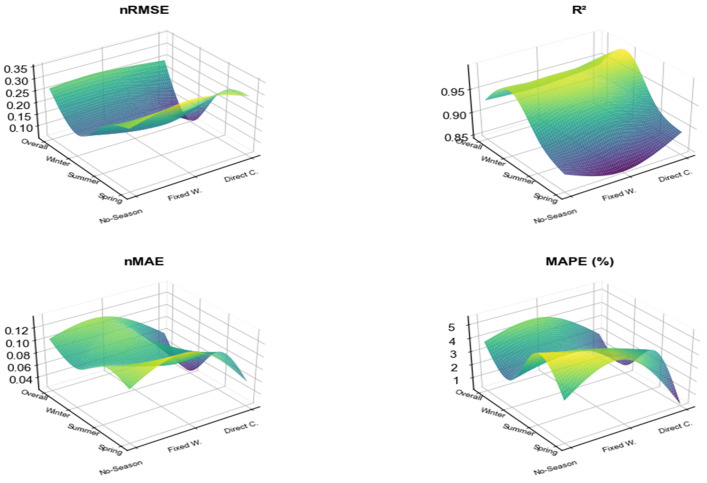
Seasonal performance comparison of fusion layer variants. In the axis labels, “No-Season” refers to the No-Season Guide variant, “Fixed W.” denotes the Fixed weight variant, and “Direct C.” denotes the Direct concat variant.

In Section 4.2.3, we designed three variants. Compared with the core model under the training settings, the experimental results are shown in Figure 14.

The core model demonstrated the best performance across the entire test set and all seasonal metrics. Direct concatenation and fusion without seasonal guidance yielded the worst results, while fixed-weight fusion performed second-worst. On the winter dataset, the core model reduced the nRMSE by 79.6%, 68.2%, and 52.3% compared to the three variants, respectively. Although the performance improvement was slightly lower in spring and summer, the core model still maintained a significant advantage.

This significant performance gap fully validates the necessity and synergy of the three core design elements in this paper’s model: the static feature branch supplements long-term patterns and seasonal variations dominated by factors such as meteorology and pollution sources; the temporal branch achieves precise capture of short-term fluctuations and long-term dependencies; and seasonal modeling adapts to the distinct pollution characteristics of different seasons. These three components mutually reinforce each other and are irreplaceable. A rigid, single-component structure or the omission of any core component would prevent the model from fully adapting to the complex characteristics of AQI prediction where temporal and static factors intertwine and seasonal variations are pronounced. By synergistically integrating multiple components, the Full-Model ultimately achieves simultaneous improvements in prediction accuracy and generalization capability.

## 6. Comparison with Other Studies

### 6.1. Comparative Analysis with the Baseline Model

**Figure 15:**
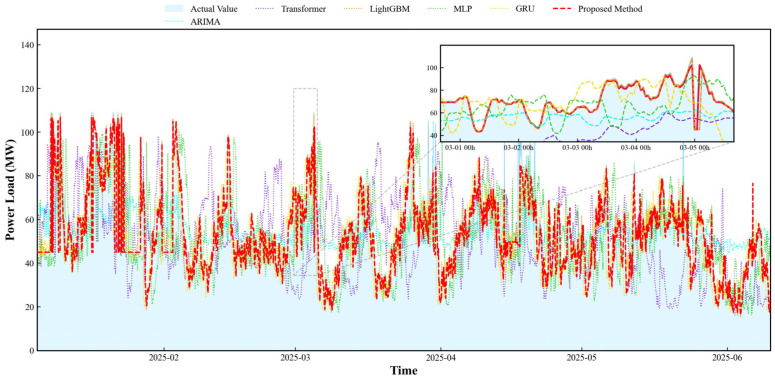
Time series prediction results of different baseline models.

**Figure 16:**
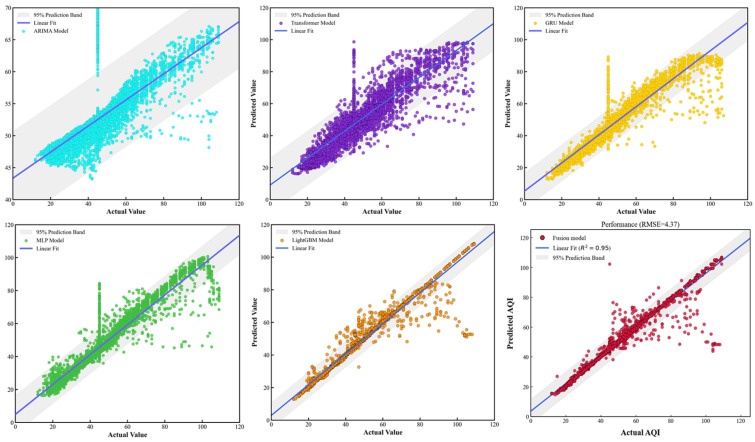
Scatter plots of predicted vs. actual values for different models.

**Figure 17:**
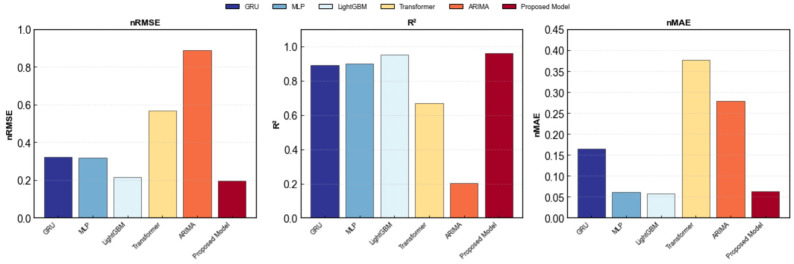
Bar chart comparison of performance metrics across different models.

To validate the superiority of the proposed prediction method over other classical approaches and clarify its core competitiveness in AQI prediction tasks, baseline model comparison experiments were designed. Five representative classical models were selected as baselines: the traditional time series model ARIMA, the complex time series deep learning model Transformer, the shallow static neural network model MLP, the ensemble learning static model LightGBM, and the classical time series deep learning model GRU. All baseline models were configured with fixed settings aligned with the core model components to ensure a fair comparison. The specific parameter settings and experimental results are shown in Table 12 and Table 13 and Figure 15, Figure 16 and Figure 17.

The selected baseline models are designed to align with typical modeling approaches for AQI prediction tasks, providing clear comparative significance: ARIMA, as the benchmark model for traditional time series forecasting, relies solely on historical AQI sequences for modeling. It serves to validate the fundamental improvements of modern fusion methods over traditional time series modeling. Transformer represents the current State-of-the-Art in complex time series modeling, leveraging self-attention mechanisms to capture long-range temporal dependencies. It is used to compare the performance boundaries between pure time series deep models and dual-branch fusion models. MLP, as a shallow static neural network, fits nonlinear relationships solely through basic features. It serves to validate the necessity of complex model structures and the fusion of temporal and static features for improving prediction accuracy. LightGBM, a high-performance ensemble learning model, excels in static feature fitting tasks and is used to compare the adaptability of a single static ensemble model versus a dual-branch fusion model in complex pollution scenarios. GRU, as a classic deep learning model for time series, serves as a representative of pure time-series deep modeling and is used to compare the performance boundaries between “pure time-series deep models” and “time-series and static” dual-branch fusion models.

Specifically, while the ARIMA model performs reasonably well on the nMAE metric, its R^2^ remains around 0.2, and its nRMSE is significantly higher than that of other models. This indicates that linear statistical methods struggle to effectively decouple complex nonlinear dependencies within the data, leading to substantial deviations in prediction results during periods of high numerical volatility.

In the deep learning baseline, the Transformer model’s performance fell short of expectations, exhibiting relatively high nMAE and nRMSE errors, with an $R^{2}$ value of only around 0.67. This may be attributed to the complex attention mechanism being prone to overfitting with limited sample sizes, thereby weakening its generalization capabilities. In contrast, GRU and MLP demonstrated strong stability, with both achieving $R^{2}$ values around 0.90. They effectively captured the main trends in the data, though some lag persisted in peak regions.

Among all baseline models, LightGBM demonstrated the most competitive performance, achieving an *R*^2^ exceeding 0.94 and significantly lower error metrics than other single models. This validates the effectiveness of ensemble tree models in reducing residuals through gradient boosting strategies. However, Scatterplot 16 reveals that despite LightGBM’s overall high fit, prediction points in the high-value range still exhibit a certain degree of divergence.

In contrast, the proposed method achieves further reductions across all error metrics, with *R*^2^ reaching 0.9611. Compared to the rule-stacked LightGBM, this architecture reserves an interface for future integration of more complex image-based meteorological data. Furthermore, the PSO-optimized fusion layer effectively mitigates the overfitting risks inherent in single deep learning models. Scatter plot 16 demonstrates that the proposed method’s predicted values closely align with actual values, clustering near the y=x diagonal. The 95% prediction band comprehensively covers the vast majority of data points. Quantitative results show the proposed method achieves an nRMSE of approximately 0.2 and maintains an *R*^2^ consistently above 0.95. Compared to the slightly inferior GRU model, the proposed method reduces nRMSE by approximately 30%, indicating not only higher prediction accuracy but also greater robustness in handling complex temporal dependencies and nonlinear features. This further validates the advantages of the fusion framework in integrating heterogeneous models, effectively overcoming the limitations of single models and demonstrating significant generalization capabilities and practical value in real-world prediction scenarios.

### 6.2. Comparative Analysis with Similar Mixed Models

In addition to the baseline model described above, this study further compares the proposed model with three hybrid models published in recent years. The selected comparison models include: the CNN-LSTM-MHA+GRU model by Sreenivasulu et al. [20], the SSA-LSTM-XGBoost model by Diaz et al. [40], and the EMD-BO-XGBoost model by Zhang et al. [41].

All three of these models rely solely on historical pollutant time-series data; they do not incorporate static features such as meteorological factors, nor are they specifically designed to address seasonal heterogeneity, and they all employ fixed-weight fusion methods. In contrast, the model proposed in this paper adopts a dual-branch parallel architecture, decoupling temporal dynamic information from static regularity information into two independent pathways. The static branch innovatively employs a seasonal XGBoost modeling strategy (training independent for spring, summer, autumn, and winter), while the fusion layer achieves dynamic weight integration through a learnable XGBoost fusion module. These architectural differences collectively constitute the source of our model’s advantages in the breadth of feature utilization and the depth of seasonal adaptation.

## 7. Conclusions

This paper proposes a hybrid prediction model (CNN-LSTM-XGBoost-Fusion) based on dual-branch feature decoupling and dynamic weighting, aiming to address the challenges of multi-source feature coupling and seasonal distribution variations in air quality index forecasting. By integrating the strengths of deep learning in temporal feature extraction with ensemble learning’s advantages in structured data regression, this method constructs a robust, high-precision forecasting framework. Empirical validation across multi-year historical meteorological and pollutant datasets confirms the model’s effectiveness in complex scenarios. Based on experimental results and analysis, the following key conclusions are drawn:

1. In order to improve the model’s ability to capture multi-scale information, this study implements temporal feature reconstruction based on window sliding and static cross-feature engineering based on domain knowledge. This strategy expands the raw input from a single dimension to a high-dimensional feature space containing local temporal dependencies (extracted by CNNs) and long-term meteorological interactions (extracted by XGBoost). Experiments show that compared with the direct input of the original data, this divide-and-conquer feature processing method significantly reduces the difficulty of nonlinear mapping and lays the data foundation for the high-precision prediction of the model.
2. In comparisons with various mainstream baseline models, including ARIMA, MLP, LightGBM, GRU, and Transformer, the proposed fusion model consistently achieved the smallest error metrics. Specifically, on the test set, it attained an nRMSE of approximately 0.197, an nMAE of approximately 0.063, and an *R*^2^ score as high as 0.96. Statistical results indicate that compared to the best-performing single baseline model, our method reduces RMSE by approximately 10% and significantly increases *R*^2^ by several percentage points. These findings robustly demonstrate the dual advantages of the proposed approach in both point prediction accuracy and trend fitting capability.
3. By constructing an ablation experiment with multiple variants from simple to complex, the key role of “seasonal modeling” and “dynamic fusion layer” is confirmed. Without simple linear superposition, the lightweight XGBoost fusion layer designed in this paper can automatically adjust the weights of temporal and static branches according to the feature distribution of different seasons. The analysis shows that the model can maintain a low level of variance in winter with severe data fluctuations or summer with complex components, and its 95% prediction band closely covers the true value, indicating that the framework effectively overcomes the lag and instability of traditional models in predicting extreme values.
4. Importance analysis and seasonal testing results demonstrate that the model exhibits excellent adaptability across different temporal scales. It maintains consistent performance across both summer datasets dominated by photochemical reactions and winter datasets characterized by particulate matter pollution. This robust cross-seasonal behavior confirms that the proposed dual-branch architecture is not only suitable for specific short-term forecasting tasks but also possesses generalization potential for long-term deployment in practical environmental monitoring systems

At the same time, this study has some limitations and areas for improvement. Future research could conduct model sensitivity analyses to systematically evaluate the impact of various hyperparameters on prediction stability and identify the key parameters that most significantly affect model robustness. Second, the framework could be extended to other cities to validate the model’s generalizability under different geographical and climatic conditions. Third, the study could explore the incorporation of additional meteorological factors and spatiotemporal features to further improve the model’s prediction accuracy during extreme pollution events.

## Figures and Tables

**Table 1:** Descriptive statistical analysis of core variables in the dataset.

Variable	Mean	Std	Min	Q1	Median	Q3	Max	Skewness	Kurtosis
AQI	47.2	20.4	9	32	45	58	109	0.83	0.33
PM_2.5_ (μg/m^3^)	32.2	20.3	0	17	26	41	106	1.31	1.32
PM_10_ (μg/m^3^)	50.3	27.9	0	30	43	64	156	1.15	1.01
O_3_ (μg/m^3^)	44.4	31.8	3	21	37	59	148	1.19	0.91
NO_2_ (μg/m^3^)	28.7	11.9	5	20	28	36	62	0.53	−0.26
Temperature (°C)	19.5	8.4	−1.8	12.3	19.5	25.9	43.3	0.14	−0.80
Precipitation (mm)	0.14	0.52	0	0	4 × 10^−4^	0.05	13.5	8.94	122.4

**Table 2:** Overall and seasonal performance metrics of the proposed model on the training set.

	nRMSE	R^2^	nMAE	MAPE
Overall	0.084	0.9930	0.063	3.30%
Spring	0.086	0.9926	0.063	2.65%
Summer	0.068	0.9902	0.054	3.48%
Autumn	0.083	0.9907	0.063	3.99%
Winter	0.106	0.9919	0.080	2.44%

**Table 3:** Overall and seasonal performance metrics of the proposed model on the validation set.

	nRMSE	R^2^	nMAE	MAPE
Overall	0.198	0.9785	0.088	3.96%
Spring	0.154	0.9926	0.081	3.23%
Summer	0.243	0.8730	0.087	4.40%
Autumn	0.246	0.9967	0.105	5.65%
Winter	0.103	0.9919	0.078	2.42%

**Table 4:** Overall and seasonal performance metrics of the proposed model on the test set.

	nRMSE	R^2^	nMAE	MAPE
Overall	0.251	0.9368	0.098	3.67%
Spring	0.304	0.8838	0.104	3.26%
Summer	0.212	0.9382	0.102	5.16%
Autumn	0.108	0.9913	0.071	1.86%
Winter	0.251	0.9368	0.098	3.67%

**Table 5:** Hyperparameter tuning ranges and optimal values for this model.

Module	Hyperparameters	Search Range	Optimal Value
Temporal Branch (CNN-LSTM)	Number of LSTM units	[32, 256]	106
	LSTM learning rate	[0.001, 0.01]	0.00484
	Number of CNN convolutional layers	[16, 64]	32
	Size of CNN convolutional layers	[3, 5]	3
	Batch size	[32, 128]	64
Static branching (seasonal XGBoost)	Number of trees	[50, 300]	144
	Maximum tree depth	[3, 8]	4
	Learning rate	[0.01, 0.3]	0.05
Fusion Layer(XGBoost)	Number of trees	[50, 150]	78
	Maximum tree depth	[3, 6]	5
	Learning rate	[0.01, 0.2]	0.05

**Table 6:** Test set performance metrics of the proposed model after PSO parameter tuning by season.

	nRMSE	R^2^	nMAE	MAPE
Overall	0.197	0.9611	0.063	2.43%
Spring	0.260	0.9169	0.085	2.86%
Summer	0.150	0.9528	0.071	4.24%
Winter	0.045	0.998	0.024	0.79%

**Table 7:** Spring results of each variant in the ablation experiment.

Season	Models	nRMSE	R^2^	nMAE
Spring	Single-LSTM	0.346	0.8774	0.159
	No-Season XGBoost	0.283	0.8999	0.063
	Single CNN-LSTM	0.302	0.8845	0.145
	Single CNN	0.448	0.7457	0.260
	Dual-branch	0.293	0.8915	0.104
	No-Season Guide	0.307	0.8811	0.105
	Fixed weight	0.350	0.8460	0.134
	Direct concat	0.292	0.8920	0.059
	Season-Encoded XGBoost	0.30	0.8899	0.097
	Full-Model	0.260	0.9169	0.085

**Table 8:** Summer results of each variant in the ablation experiment.

Season	Models	nRMSE	R^2^	nMAE
Summer	Single-LSTM	0.260	0.9276	0.144
	No-Season XGBoost	0.215	0.9368	0.071
	Single CNN-LSTM	0.211	0.9387	0.123
	Single CNN	0.355	0.8275	0.241
	Dual-branch	0.208	0.9408	0.100
	No-Season Guide	0.240	0.9208	0.113
	Fixed weight	0.174	0.9585	0.088
	Direct concat	0.237	0.9231	0.081
	Season-Encoded XGBoost	0.181	0.9258	0.075
	Full-Model	0.150	0.9528	0.071

**Table 9:** Winter results of each variant in the ablation experiment.

Season	Models	nRMSE	R^2^	nMAE
Winter	Single-LSTM	0.419	0.771	0.216
	No-Season XGBoost	0.020	0.9997	0.013
	Single CNN-LSTM	0.564	0.7504	0.291
	Single CNN	0.974	0.2567	0.797
	Dual-branch	0.143	0.9839	0.091
	No-Season Guide	0.141	0.9844	0.084
	Fixed weight	0.139	0.9858	0.107
	Direct concat	0.051	0.9979	0.023
	Season-Encoded XGBoost	0.067	0.9956	0.037
	Full-Model	0.045	0.998	0.024

**Table 10:** Overall results of each variant in the ablation experiment.

Models	nRMSE	R^2^	nMAE
Single-LSTM	0.337	0.883	0.170
No-Season XGBoost	0.235	0.9447	0.057
Single CNN-LSTM	0.335	0.8843	0.161
Single CNN	0.545	0.6941	0.341
Dual-branch	0.246	0.9378	0.100
No-Season Guide	0.264	0.9286	0.104
Fixed weight	0.272	0.9258	0.114
Direct concat	0.249	0.9365	0.061
Season-Encoded XGBoost	0.232	0.9461	0.073
Full-Model	0.197	0.9611	0.063

**Table 11:** Seasonal prediction results of the ablation variant without seasonal submodels.

Season	nRMSE	R^2^	nMAE	MAPE
Spring	0.293	0.8915	0.104	3.28%
Summer	0.208	0.9408	0.100	5.03%
Winter	0.143	0.9839	0.090	2.29%
Overall	0.246	0.9378	0.100	3.72%

**Table 12:** Hyperparameter tuning for the baseline model in this paper.

Model	Key Parameter	Value	Description
ARIMA	Order	(2, 1, 2)	Fixed based on autocorrelation analysis
Transformer	Number of layers/channels/dimensions	2 layers/4 channels/32 dimensions	Standard configuration
MLP	Hidden layer structure	64 → 32	Aligned with the static branches in this paper
LightGBM	num_leaves/lr/estimators	64/0.05/100	Common default values
GRU	Number of hidden units/Dropout	64/0.2	Align with the timeline in this article

**Table 13:** Comparative performance metrics of the proposed model and baseline models.

Models	nRMSE	R^2^	nMAE
GRU	0.324	0.8921	0.164
MLP	0.319	0.898	0.061
LightGBM	0.216	0.9533	0.058
Transformer	0.567	0.6673	0.377
ARIMA	0.890	0.2022	0.279
Proposed Model	0.197	0.9611	0.063

## Data Availability

Data will be made available on request.
